# Optimization of periodic treatment strategies for bacterial biofilms using an agent-based *in silico* approach

**DOI:** 10.1098/rsif.2024.0078

**Published:** 2024-04-10

**Authors:** Johanna A. Blee, Thomas E. Gorochowski, Sabine Hauert

**Affiliations:** ^1^ School of Engineering Mathematics and Technology, University of Bristol, Ada Lovelace Building, Tankard's Close, Bristol BS8 1TW, UK; ^2^ School of Biological Sciences, University of Bristol, Life Sciences Building, 24 Tyndall Avenue, Bristol BS8 1TQ, UK; ^3^ BrisEngBio, School of Chemistry, University of Bristol, Cantock's Close, Bristol BS8 1TS, UK

**Keywords:** biofilms, persister, antibiotic regimen, agent-based modelling

## Abstract

Biofilms are responsible for most chronic infections and are highly resistant to antibiotic treatments. Previous studies have demonstrated that periodic dosing of antibiotics can help sensitize persistent subpopulations and reduce the overall dosage required for treatment. Because the dynamics and mechanisms of biofilm growth and the formation of persister cells are diverse and are affected by environmental conditions, it remains a challenge to design optimal periodic dosing regimens. Here, we develop a computational agent-based model to streamline this process and determine key parameters for effective treatment. We used our model to test a broad range of persistence switching dynamics and found that if periodic antibiotic dosing was tuned to biofilm dynamics, the dose required for effective treatment could be reduced by nearly 77%. The biofilm architecture and its response to antibiotics were found to depend on the dynamics of persister cells. Despite some differences in the response of biofilm governed by different persister switching rates, we found that a general optimized periodic treatment was still effective in significantly reducing the required antibiotic dose. As persistence becomes better quantified and understood, our model has the potential to act as a foundation for more effective strategies to target bacterial infections.

## 1. Introduction

Antimicrobial resistance (AMR) has been declared by the World Health Organization as one of the top 10 global public health threats facing humanity [1]. They have estimated that by 2050 it will cause 10 million deaths per year and cost the global economy $100 trillion if not addressed. Antibiotic overuse can increase AMR and causes harmful side effects.

Most clinical infections are not caused by planktonic bacteria, but by communities of cells known as biofilms [2]. Biofilms are highly tolerant to antibiotics requiring 100–10 000 times the levels than cells growing planktonically [3]. One of the key reasons biofilms are so difficult to eradicate is phenotypically persistent subpopulations [4,5].

Contrary to resistant cells, persister cells do not exhibit genetic changes and are not able to withstand higher concentrations of antibiotic. They instead endure treatments for extended periods due to phenotypic changes [6]. Persistence is a generalized and transient mechanism of survival enabling protection against a variety of stresses and biocides [7]. It is a spontaneous and reversible phenotypic change that is characterized by slow growth and increased survival time. Persistent subpopulations can survive antibiotic treatments before switching back to a susceptible state, allowing the biofilm to survive and recover from antibiotic treatments [4,5]. The transient and rapid dynamics associated with persistence make it a powerful strategy in changing environments. Persistence has been associated with a range of chronic and recurrent infections, such as tuberculosis, urinary tract infection and cystic fibrosis [8–10].

The mechanisms and dynamics that govern cells switching from a susceptible to a persister state and back again are highly dependent on the environment and the specific strain of cell [11–15]. It has been shown that persister populations increase in stationary phase and during biofilm formation. The mechanisms underlying persistence are still under debate; however, two distinct types of persisters have been identified. Triggered persisters, which form in response to stress, and stochastic persisters, which have been observed without any trigger and are far less common [7].

Persistence levels are dependent on environmental conditions, such as nutrient and oxygen availability, pH and presence of biocides [4,5,16]. The persister switching dynamics may be highly variable and is often linked not just to the specific environmental conditions but also to previous conditions that cause the biofilm to evolve over time [11,17]. The mechanisms underpinning persistence are still heavily debated, but persistence has been linked to a lack of growth and to toxin-antitoxin (TA) modules [11,18]. It has been proposed that TA pairs induce a state of dormancy and increase tolerance, and several TA systems have been linked to persistence [18–20]. However, some bacteria appear to form persisters dependent on ATP levels or metabolic genes instead of TA modules [21].

Regardless of the mechanisms involved in persistence, when the levels of antibiotic are above the minimum inhibitory concentration (MIC) biphasic killing curve is observed. The first part of the curve is characterized by the susceptible cell death rate and the second part by the slower persister death rate. Persistence appears to be consistent across antibiotic levels (10–500 times the MIC) [22]. Therefore, if the treatment is not applied for long enough the persister cells will survive and switch back into a susceptible state, causing the infection to regrow [5,23]. Persistence has also been linked to resistance [13,17,24–26]. It is therefore paramount that dosing regimens fully eliminate persister subpopulations.

It has been shown that periodic dosing can be used to reduce the total effective antibiotic dose by ‘reawakening’ the persistent subpopulations to make them more susceptible to treatment [27–31]. Sustained exposure to antibiotics poses an increased risk of resistance development in not just the targeted but also surrounding bacterial population. It can also have negative side effects on the patient, including damage to the microbiome. By reducing antibiotic exposure while effectively treating persistent subpopulations periodic dosing could minimize patient side effects as well as the emergence of antibiotic resistance.

The inherent heterogeneity of biofilms, along with the diverse dynamics of persister cells and biofilm structures, gives rise to varied responses of biofilms to antibiotic treatments [3,4,27,32–34]. Computational models can be used to simulate a wide range of scenarios rapidly and cheaply to find optimal treatment schedules that can be tested and verified *in vitro* and *in vivo*.

Heterogeneity in biofilms, from the phenotypic state of cells to environmental conditions, affect the biofilm architecture, the location of persister cells and the response to antibiotic treatments [4,16,35]. Several computational models of biofilms have been developed to examine different persister switching mechanisms and rates [29,36–41]. Most of these rely on differential equations, which are deterministic, do not consider detailed spatial variations and have continuous collective responses built in. They therefore fail to capture the stochasticity, heterogeneity and collective emergent behaviour that is intrinsic to biofilms. Agent-based models can better capture these characteristics and have become increasingly popular for studying cellular populations and biofilms [42–44].

Several agent-based models have been used to demonstrate the importance of the spatial organization of persister cells and how the physical architecture of the biofilm, survival and recovery from antibiotics, depend on the persistence switching mechanism [35,38,45,46]. For example, one study used agent-based models to explore different types of persister switching, random, nutrient dependent and antibiotic dependent [35]. They found that the location of persisters in the biofilm was linked to the persistence mechanism and that this affected the biofilms architecture following recovery. Certain architectures may be beneficial to survival, for example susceptible cells near the surface may offer a competitive advantage in polymicrobial biofilms or in detachment and future colonization [47].

Unfortunately, current models often lack two crucial features. Firstly, some do not include the switching from persister to susceptible state, meaning that recovery cannot be tested. Secondly, many only consider distinct antibiotic-dependent or substrate-dependent switching, even though it has been shown that persister formation in biofilms is dependent on both antibiotic presence and substrate availability [48–50].

**Figure 1.**
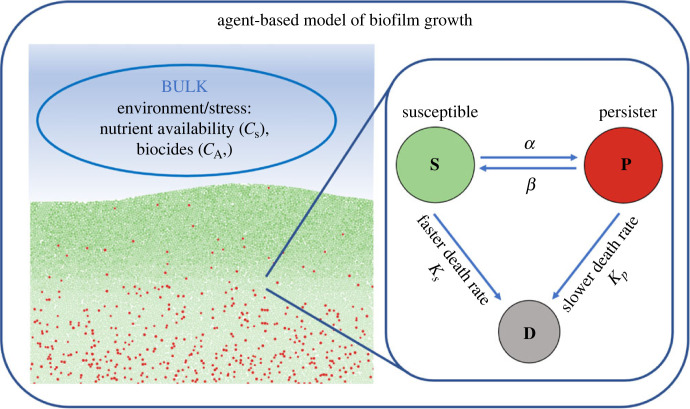
Our agent-based *in silico* model of a biofilm consisting of susceptible cells, persister cells, dead cells, substrate and antibiotic. Each bacterium in the biofilm is considered individually and their behaviour depends on the local environment and cells surrounding them. The rate at which susceptible cells switch to the persister state (α) is lower under low stress and higher during high stress. Contrastingly, the rate at which persister cells switch back to the susceptible state (*β*) is low under high stress and high under low stress. Susceptible cells die at a faster rate when exposed to antibiotics than persister cells.

In this work, we address these limitations by developing an agent-based model of biofilm growth and persister formation that incorporates spatial and temporal heterogeneity, as well as more realistic switching mechanisms (figure 1). It has been shown that persister formation in biofilms is dependent on both antibiotic presence and substrate availability, and so in a similar way to previous differential equation-based models we incorporated these switching mechanisms into an agent-based model (figure 1). Our model is based on the model of Carvalho *et al.* [35], but with added persister dynamics and the addition of periodic dosing.

Using our model, we examined how biofilms with different persister switching dynamics responded to antibiotic treatments. We also explored how periodic dosing could be used to reduce the dose of antibiotics while still effectively treating the whole biofilm. We show that periodic dosing aligned to a biofilm's dynamics could significantly reduce the required dose of antibiotics. These findings and the model itself provide valuable insight and tools for further refinement of treatment approaches that overcome difficulties arising from persistence in biofilms.

Several different strategies are currently being explored to target persisters. These include the use of anti-persister drugs, inhibiting persister formation and resensitizing persistent subpopulation [34,51–53]. It is likely that a combination of strategies will be required to tackle persistent infections. We have focused on dosing regimens as a simple first step as we develop new treatments and gain understanding on the mechanisms underpinning persistence. Optimized dosing regimens offer us more efficient and effective ways of deploying existing drugs and can be integrated with forthcoming anti-persister strategies.

## 2. Material and methods

### 2.1. Agent-based model

**Figure 2.**
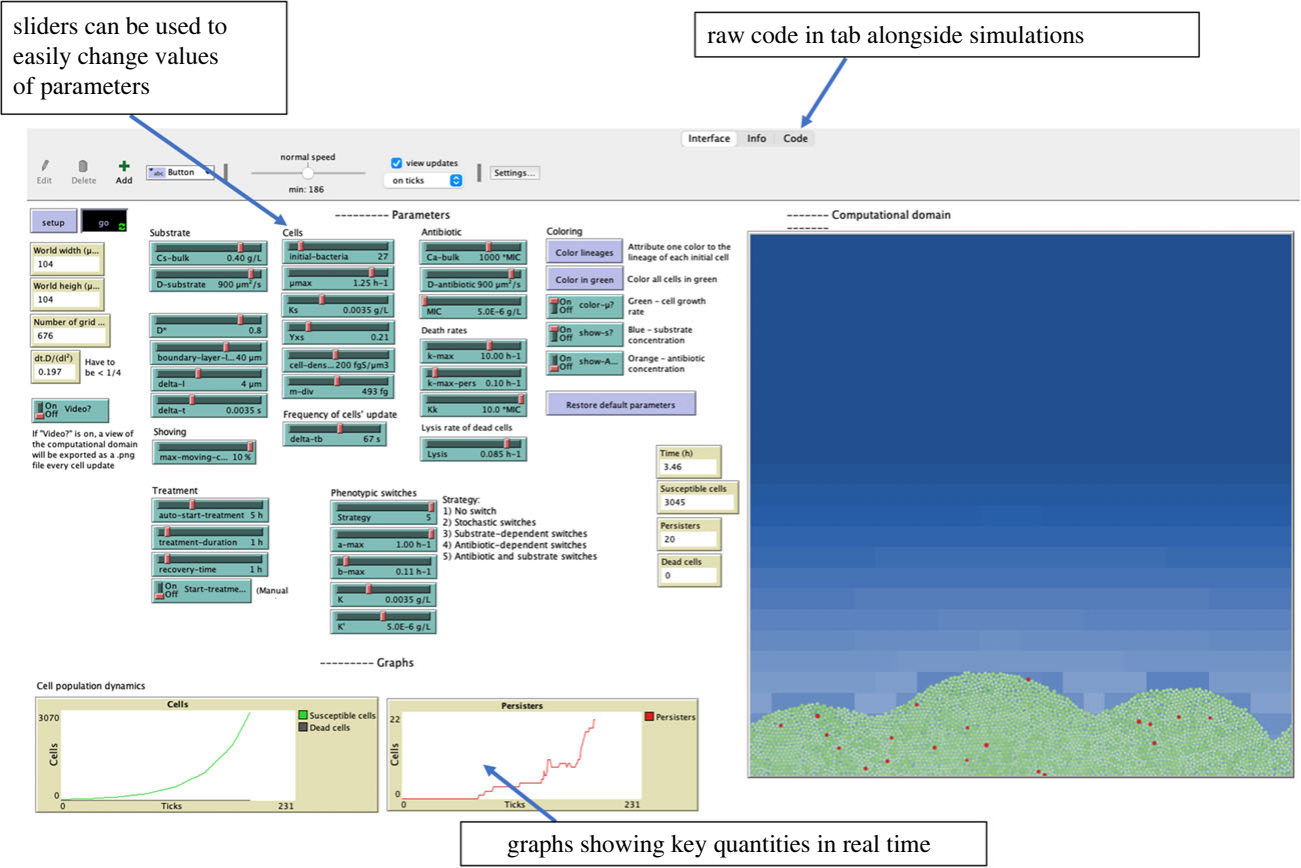
Netlogo's user interface showing our agent-based model. The sliders and toggles can be used to adjust model parameters, the simulations can be viewed alongside these along with graphs showing key quantities, such as the number of different cell types in real time. The raw code which contains the model is also available and can be edited in an adjoining tab.

We developed a computational two-dimensional agent-based model of biofilm growth and persister formation. This was used to simulate different dynamics and antibiotic treatment regimens to find optimized treatments. Our model builds on previous agent-based biofilm models [35,44,54]. Cells in a biofilm grow and divide depending on the quantity of local substrate available to them and a ‘shoving’ algorithm is applied to resolve overlapping cells. Cells in a biofilm can switch to a persister state and back again. The switching rates are dependent on the quantity of substrate and the presence of antibiotics. Susceptible and persister cells are killed by the antibiotic at different rates. The substrate and antibiotic both diffuse from the bulk liquid above the biofilm. The concentrations of the antibiotic and substrate and the individual cell states are updated at each time step in the simulation. As with previous models it is assumed that the total simulation time is short enough to ignore detachment, cell maintenance, shrinking of the biofilm and extracellular polymeric substances (EPS) production [35]. Our model was implemented in NetLogo [55]. This facilitated the addition of a graphical interface, which allows users to easily change model parameters using sliders and toggles and visualize the effect these chances have on biofilm growth dynamics (figure 2). Because it is computationally expensive to run simulations with the graphical interface, we also provide access to a command-line version which executes large models faster.

### 2.2. Biofilm growth

Biofilm growth was initialized in our simulations by setting 27 susceptible bacteria randomly on the surface. This number of cells was found to be appropriate for our surface size. Initialization mimicked the process seen in natural biofilms with several cells adhering randomly across the surface. The cells grew following a Monod kinetic model. The growth of susceptible cells was related to the local availability of the substrate (*C_S_*) and the mass (*m_i_*) of a susceptible cell *i* was given by

2.1 $$\frac{dm_{i}}{dt}=m_{i}\mu_{\max}\frac{C_{S}}{C_{S}+K_{S}},$$

where *μ*_max_ is the maximal specific growth rate and *K*_S_ is the half-saturation constant. Each cell was modelled as a cylinder with a length of *l* and diameter *d_i_* and density *ρ* related by

2.2 $$d_{i}=\sqrt{\frac{4m_{i}}{\rho l\pi }}.$$

Cell division occurs at a threshold mass (default 500 fg) when the mass of the mother cell is split randomly 40–60% to create two daughter cells [56]. The position of the daughter cells is randomly directed with zero overlap equidistant from the mother's centre. To prevent cells overlapping during growth a shoving algorithm is applied. The shoving vector *u_S_*_,*i*_ moves an overlapping cell *i* away from its neighbour towards its own centre and is calculated using

2.3 $$u_{S,i}=\sum_{h=1}^{N}\frac{a_{i}+a_{h}-d_{h}}{2}\cdot u_{h},$$

where *a_i_* is the cell radius, *a_h_* is the radius of the neighbour, *d_h_* is the distance between the centre of the two overlapping cells and *u_h_* is a unitary vector directed towards the centre of the cell from the centre of the neighbour. All shoving movements are applied simultaneously and continued until less than 5% of the agents are still moving or until 1000 iterations per cell are applied.

### 2.3. Diffusion-reaction of substrate and antibiotics

The antibiotic and substrate concentrations (*C*_A,S_) both obey the diffusion-reaction equation,

2.4 $$\frac{dC_{A,S}}{dt}=D_{A,S}\cdot \nabla \cdot C_{A,S}+r_{A,S},$$

where *D*_A,S_ is the antibiotic or substrate biofilm diffusion coefficient. Both are assumed to be 80% of the diffusion coefficients in water and *r_A_*_,*S*_ are the consumption/production terms. These are calculated at each time step over a set of predefined grids of length Δ*l* using a finite-element model. The diffusion-reaction of the substrate and antibiotic are discretized into finite-elements and solved at each time step Δ*t* to give the antibiotic *C*_A_ or substrate concentration *C*_s_ at a given time point *t* and position (*x*, *y*) [35,54]. We assume that antibiotic production and consumption is zero *r*_A_ = 0 [35]. The substrate consumption/production rate *r*_S_ in a grid cell is the sum of uptake by susceptible cells in that area and the release by dead cells and given by

2.5 $$r_{S}=-M_{S,x,y}\mu_{\max}\cdot \frac{C_{S}}{Y_{x,y}(C_{S}+K_{S})}+M_{\mathrm{dead},x,y}R_{L},$$

where *M_S_*_,*x*,*y*_ and *M*_dead,*x*,*y*_ are the biomasses of the susceptible and dead cells in the grid (*x*, *y*), *Y_x_*_,*y*_ is the biomass yield (a measure of the susceptible cells growth efficiency) and *R*_L_ is the lysis rate of dead cells. Concentrations are kept constant in the bulk liquid above the biofilm and then diffuse across a boundary layer (*l*_B_ set to default of 40 nm) towards the biofilm.

### 2.4. Killing of cells

In this study, we are interested in optimizing dosing regimens for antibiotic treatments with concentrations above the MIC and so consistent with previous studies concentrations of 1000 times MIC were used [35,40]. This ensured there is biphasic killing of susceptible and then persister cells. The killing rates of susceptible *k*_S_ and persister cells *k*_P_ are given by,

2.6 $$k_{S}=k_{\mathrm{maxS}}\frac{C_{A}}{C_{A}+K_{A}}$$

and

2.7 $$k_{P}=k_{\mathrm{maxP}}\frac{C_{A}}{C_{A}+K_{A}},$$

where *k*_maxS_ and *k*_maxP_ are the maximum killing rates of the susceptible and persister cells respectively, and the half-saturation constant *K_A_* is fitted to ensure that, when antibiotic concentration equals the MIC, the death rate equals the maximal growth rate *μ*_max_. When a cell dies it remains in the simulation but is inactive and its diameter is reduced by 20%. These cells are then lysed at rate *R_L_* until a minimal critical size is reached, at which point it is removed from the simulation. As our model is an agent-based model, each cell is considered individually, and rates of cell death and persistence were converted into individual probabilities for each cell depending on its local environment. To convert rates into individual probabilities at each time step a random number between 0 and 1 was generated, and if this number was less than the rate multiplied by the time step then the action takes place.

### 2.5. Persister and susceptible cell formation

The exact mechanism of persister formation varies and is dependent on the environmental conditions. However, persister cells have been shown to form due to substrate depletion and due to the presence of antibiotics. We assume a switching model that is dependent on both the substrate availability and the presence of antibiotics. The switching rates from susceptible to persister and persister to susceptible due to substrate availability (*α*(*C*_s_), *β*(*C*_S_)) and antibiotics (*α*(*C*_A_), *β*(*C*_A_)) were given respectively as

2.8 $$\;\alpha (C_{s})\;=\alpha_{\max}\left(1-\frac{C_{S}}{C_{S}+K_{S}}\right),\;\alpha (C_{A})\;=\alpha_{\max}\left(\frac{C_{A}}{C_{A}+K_{A}}\right)\;$$

and

2.9 $$\beta (C_{s})=\beta_{\max}\left(\frac{C_{S}}{C_{S}+K_{S}}\right),\beta (C_{A})=\beta_{\max}\left(1-\frac{C_{A}}{C_{A}+K_{A}}\right).$$

For an individual cell the probability of switching was dependent on both the local antibiotic and substrate concentrations. For each susceptible cell at every time step (Δ*t*_cell_) there is a probability that it may switch to the persister state, and for each persister cell that it may switch to the susceptible state. For each susceptible cell a random number is generated between 0 and 1. If the number is larger than *α*(*C*_s_) × Δ*t*_cell_ or *α*(*C*_A_) × Δ*t*_cell_ then the cell switches to a persister. For each persister cell the same process is followed at each time step but seeing if the random number is larger than *β*(*C*_s_) × Δ*t*_cell_ or *β*(*C_A_*) × Δ*t*_cell_. The maximal switching rate *a*_max_ from susceptible to persister is reached when antibiotic levels are high (i.e. *C*_A_ ≫ MIC) and/or the substrate concentrations are low. Whereas when antibiotics are absent and there is plenty of substrate, the switching rate becomes 0. The reverse is true for switching back from a persister to susceptible state so that maximum switching *β*_max_ occurs when the substrate is high and the antibiotic absent.

### 2.6. Antibiotic treatments

Continuous or periodic antibiotic treatments were applied after 5 h of biofilm growth. Periodic treatments consist of periods of treatments of duration *t*_dur_ separated by periods of recovery of length *t*_rec_.

### 2.7. Model parametrization and sensitivity analysis

We ran simulations for a wide range of biofilms, dynamics and treatments. This was performed using NetLogo's software tool BehaviourSpace that has been designed to perform parameter scans. Due to the computational demands of these simulations, they were performed on the University of Bristol's High Performance Computing facility (HPC).

The parameters used for this study were obtained from previous studies where available (table 1). The variance and paucity in persister switching dynamics led us to explore a wide range of different switching rates. To simulate biofilms with a wide range of switching rates global sensitivity analysis was run for *α*_max_ = [0.1, 1] and *β*_max_ = [0.1, 1]. For each set of parameters, a biofilm was grown, and continuous and periodic antibiotic treatments tested. The periodic treatment duration times *t*_dur_ and recovery times *t*_rec_ were varied in the range of 10–90 min. This range was chosen, as any lower values would be difficult to implement *in vitro* and *in vivo* and 90 min was chosen as initial tests showed this would be the upper optimized limit for any parameter combinations. The periodic timings of this study are the times that the bulk above the biofilm changes and do not account for any other pharmacokinetics of substrate and antibiotic delivery *in vitro* or *in vivo*.

**Table 1.** Parameters for the biofilm model.

parameter	description	value	reference
*lx*	width of domain	104 mm	[35]
Δ*l*	length and width of grid cell	4 mm	[35]
*l*_B_	boundary length	40 mm	[54]
Δ*t*_diff_	time step for diffusion-reaction	3.5 ms	this study
Δ*t*_cell_	time step for cell updates	67 s	this study
*C*_s,bulk_	solute (glucose) concentration in bulk	0.4 g l^–1^	[35]
*D*_s_	diffusion coefficient of solute in water at 37^o^C	900 mm^2^ s^–1^	[57]
*C*_A,bulk_	concentration of antibiotic in bulk	1000 × MIC	[35]
*C*_A,MIC_	antibiotic minimum inhibitory concentration	0.05 mg l^–1^	[40]
*D*_A_	diffusion coefficient of antibiotic in water at 37^o^C	900 mm^2^ s^–1^	[40]
*μ*_max_	maximal growth rate	1.25 h^–1^	[40]
*K*_s_	half-saturation constant for the substrate	10^−5^ g l^–1^	[40]
*Y_X_*_,*Y*_	yield from susceptible cells	1187 fg cell^–1^	[40]
*d_X_*	density of cells	200 fg μm^–3^	[58]
*m*_div_	mass at which cells divide	500 fg	[58]
*k*_max_	maximum killing rate of susceptible cells	10 h^–1^	[35]
*k*_max*p*_	maximum killing rate of persister cells	0.1 h^–1^	[35]
*K*_k_	half-saturation constant for the killing rates	6.4 g l^–1^	[35]
*R*_L_	lysis rate of dead cells	0.05 h^–1^	[35]

The antibiotic dose is defined by multiplying antibiotic concentration by exposure time. For our study, the antibiotic concentration is constant and so the total antibiotic dose and exposure time can be used as equivalent measures of antibiotic exposure. Each of the continuous antibiotic treatments and periodic treatments simulations were run until all the cells were killed to give the total required antibiotic treatment time. Due to stochasticity a given set of parameters were run three times and the average and standard error (s.e.) obtained (reported as ± values in the results). Simulations were run until all cells were dead.

## 3. Results

**Figure 3.**
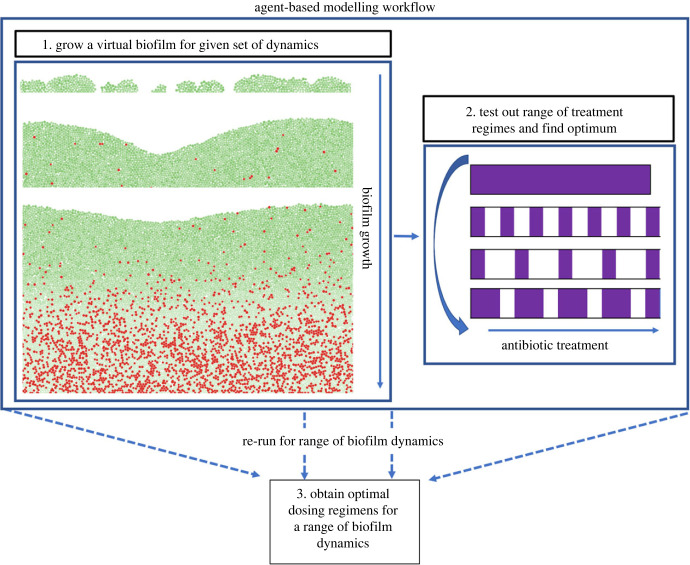
*In silico* modelling workflow. A given biofilm with given set of dynamics is grown up and then a given treatment is implemented. Treatments were either continuous or periodic. This was then repeated for a range of treatments and biofilm dynamics. Simulations were run for each set of dynamics and scenario until all the cells were dead and the quantity of antibiotics required was calculated. Simulations were repeated three time for each set of parameters.

**Figure 4.**
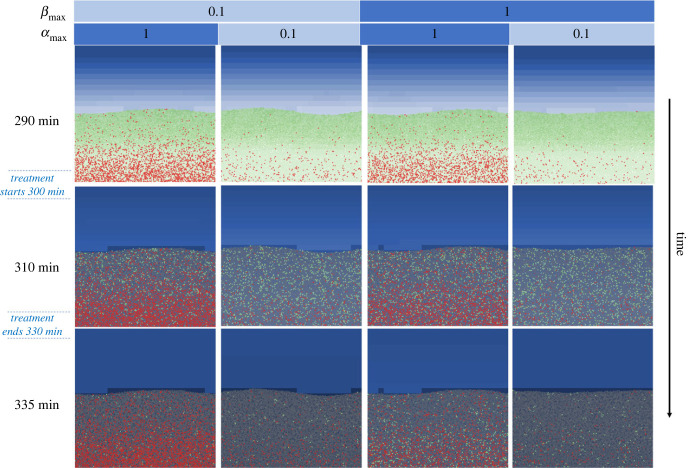
Representative time-lapse images from simulations of our agent-based *in silico* model for periodic treatment of biofilms governed by different persistence dynamics. The biofilms were treated periodically every 30 min for 30 min with antibiotics after 300 min of growth. Four simulations with different persister dynamics (*α*_max_ and *β*_max_ combinations as shown in table above) were selected as representative examples.

We developed an agent-based model of biofilm growth and persister formation (Materials and methods). This model captures spatial and temporal heterogeneity and can be used to simulate a wide range of treatment dynamics. In this study, we were interested in optimizing dosing regimens for antibiotic treatments with concentrations above the MIC. The variance and paucity of persister switching dynamics led us to explore a wide range of different switching rates. For a given set of parameters (representing specific persister and biofilm dynamics), we grow a biofilm before then exposing it to antibiotics and simulating its response. For each combination of parameters, we simulated continuous antibiotic treatments and periodic treatments (e.g. pulses of antibiotic) with a broad range of combinations of treatment and recovery times (figure 3). Over 3000 different biofilm dynamics and treatments were simulated in triplicate to help assess variability in any response. Representative examples of simulations are provided in figure 4 and electronic supplementary material, videos S1–S4.

### 3.1. Biofilm sensitization through periodic treatment

**Figure 5.**
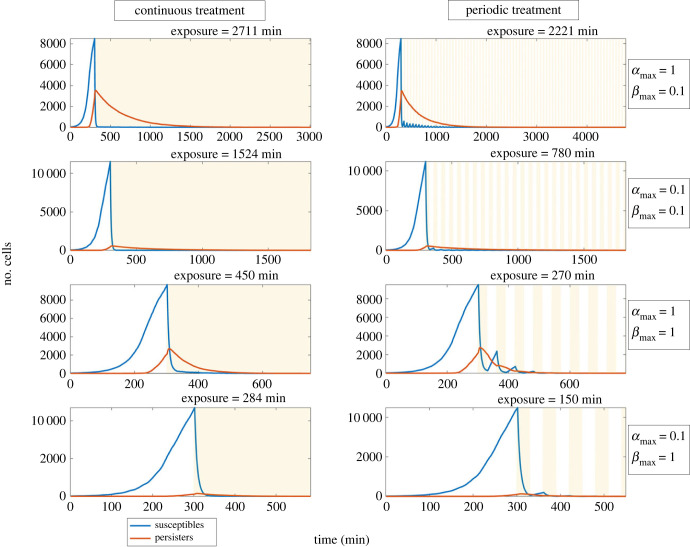
The number of susceptible cells (blue) and persister cells (red) as a function of time for different persister switching dynamics (when treated continuously or periodically (every 30 min for 30 min)). Antibiotic treatment is shown in yellow. The biofilm dynamics affect the location and quantity of persister cells which in turn affects its response to treatment.

Simulations showed that periodic dosing may be used to ‘reawaken’ persister cells and reduce the antibiotic load required to treat the biofilm. Periodic dosing consists of antibiotic treatment with durations *t*_dur_ separated by recovery periods *t*_rec_. When the levels of antibiotic were above the MIC in the whole biofilm a biphasic killing curve was generated by the killing of susceptible and persistent populations. Initially there was a large drop in cells, representing the susceptible population being killed followed by slower killing of persister cells. Persister cells take longer to be killed, and if treatment is stopped prematurely, they may switch back into a susceptible state enabling the biofilm to regrow. If instead the treatment is paused following the initial killing of susceptible cells, there is a sudden reduction in stress for the persister cells (increased access to nutrients and lack of antibiotics) and they have an increased probability of switching back to the susceptible state. If we then repeat the treatment, we can rapidly kill these cells. We found that the biofilm's dynamics affect the location and quantity of persister cells, which in turn affects its response to the treatment and recovery from it (figures 4 and 5, and electronic supplementary material, videos S1–S4). The four outer limits of persistence dynamic combinations (minimum and maximum combinations of *α*_max_ and *β*_max_ of 0.1,1) were chosen to illustrate the effect of persister dynamics on different cell populations. Figure 4 shows the number of cells over time, while figure 5 and electronic supplementary material, videos S1–S4 show corresponding time lapses. They demonstrate that the faster the switching rate from susceptible to persister state *α*_max_, and slower the switching rate back again *β*_max_, the more persisters there were prior to treatment (mainly *α*_max_ dependent) (figure 5). Whereas during the recovery phases more persisters remain if *β*_max_ is low. It is these two combined effects that contribute towards the length of time taken to treat the biofilm.

### 3.2. Optimized periodic treatment

**Figure 6.**
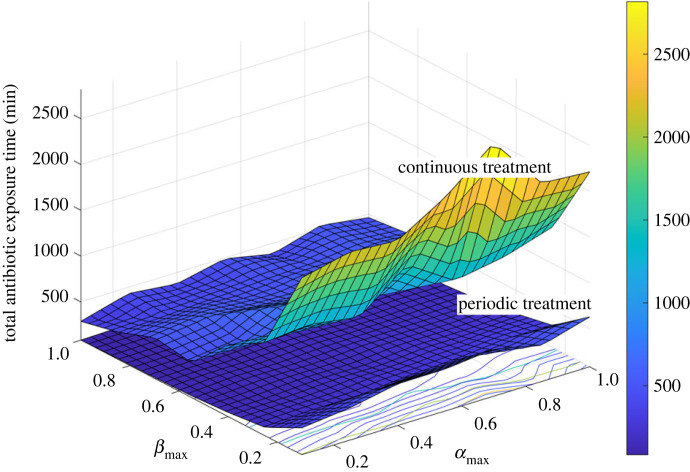
Required antibiotic exposure time as a function of persistence dynamics (*α*_max_ and *β*_max_) for continuous and optimized periodic treatments*.* Periodic treatments were optimized for each set of dynamics. Across all persister switching dynamics the optimized periodic treatments were below the continuous treatments.

**Figure 7.**
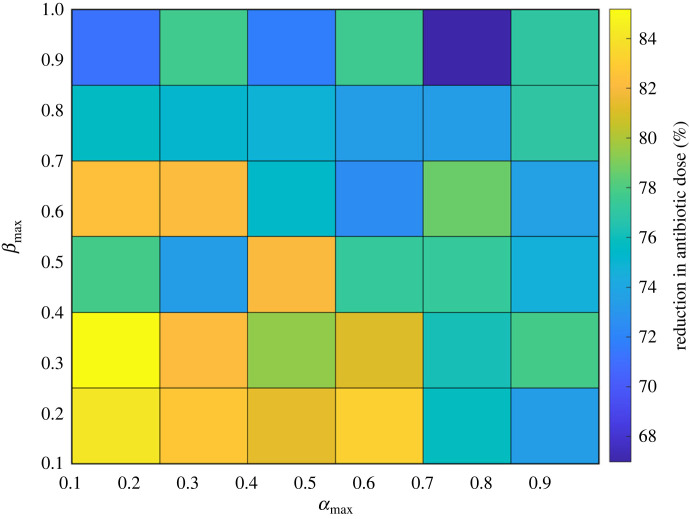
Percentage reduction in required antibiotic dose of optimized periodic treatments compared with continuous treatments as a function of biofilm persistence dynamics (*α*_max_ and *β*_max_). Periodic treatments were optimized for each set of dynamics.

We found that across the wide range of persister switching rates tested, optimizing the periodic dosing dynamics to align with the biofilm dynamics reduced the total required dose of antibiotics by 76.9 ± 0.6% (figure 6). From now on we will refer to these treatments as optimized periodic treatments. Using the percentage reduction in required antibiotic as a measure of effectiveness, optimized treatments were most effective at low switching rates to and from the persistent state (low *β*_max_ and *α*_max_) and least effective at higher switching rates (high *β*_max_ and *α*_max_) (figure 7).

The optimal treatment and recovery times of treatments depended on the persistence dynamics (electronic supplementary material, figure S1). At high persistence switching rates (high *β*_max_ and *α*_max_) shorter treatment durations and recovery periods were optimal. Whereas at low switching rates the optimal periodic treatments were composed of the longest treatment durations and recovery periods.

**Figure 8.**
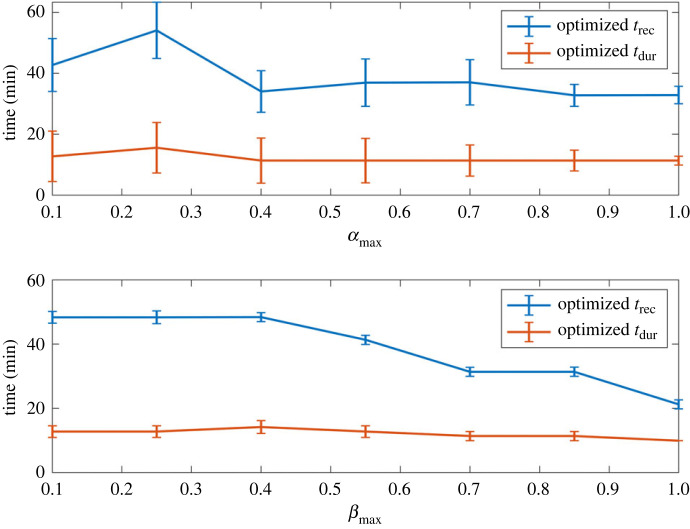
Optimized treatment duration and recovery times (*t*_dur_ and *t*_rec_) as a function of persistence dynamics. The top figure shows optimized treatments as a function of *α*_max_ averaged over all *β*_max_ and the bottom figure shows the optimized treatments as a function of *β*_max_ averaged over all *α*_max_. The errors show the standard deviation.

The variation in the optimal recovery time (*t*_recop_) was far greater than the change in the optimal treatment duration (*t*_durop_) across different persister switching dynamics (figure 8). Optimal treatment duration and recovery time were strongly linearly correlated (Pearson's correlation coefficient = 0.88). The optimal duration was always smaller than the optimal recovery time with the mean and standard deviations for *t*_dur_ and *t*_rec_ across all dynamics being 12 ± 4 min and 39 ± 19 min respectively. For all 81 persistent dynamics tested only eight optimized treatment strategies emerged given by [*t*_dur_, *t*_rec_ (min)] = [10, 20], [10, 30], [10, 40], [20, 50], [20, 60], [20, 70], [20, 80], [20, 90].

### 3.3. Generalized optimal periodic treatments

**Figure 9.**
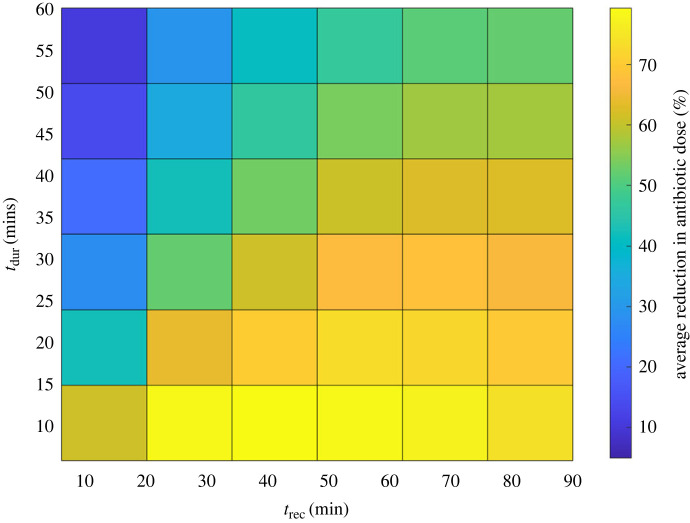
Percentage reduction in antibiotic exposure time as a function of periodic treatment duration and recovery times (*t*_dur_ and *t*_rec_) averaged over all persister switching dynamics (*α*_max_ and *β*_max_ combinations).

Persistence mechanisms and dynamics are not fully understood and can vary. It is also not currently possible to monitor them dynamically *in vitro*. It would therefore be beneficial to design generalized optimized strategies that are effective against a range of persistence dynamics. From now on we will refer to these as generalized optimized periodic treatments. Figure 9 shows the average percentage reduction in antibiotics for each tested treatment strategy (*t*_dur_ and *t*_rec_ combination) across all persister dynamics (*α*_max_ and *β*_max_ = [0.1, 1]). This landscape can be used to assess how effective a given set of treatment dynamics is as a generalized strategy (against all persister switching dynamics). An alternative measure of generalized effectiveness is the average fitness of a given strategy across all persister dynamics compared with the optimum for those dynamics. In this case, we found that the two strategies matched (electronic supplementary material, figure S2). Figure 9 and electronic supplementary material, figure S2 show that the specific optimum periodic treatment regimens identified in the previous section, were also the general optimums.

**Figure 10.**
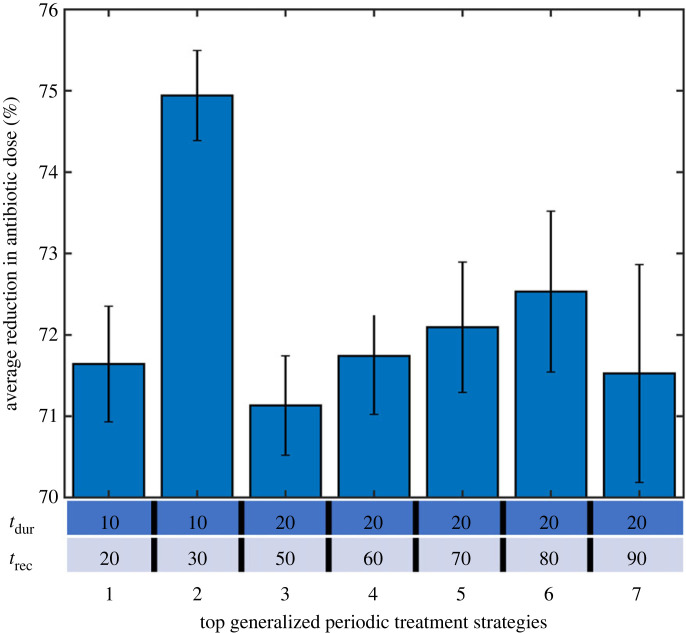
Percentage reduction in required antibiotic dose for the top generalized optimized treatment strategies (1–7) averaged across all tested biofilm persistent dynamics (*α*_max_ *and β*_max_ *= 0.1–1*). The treatment dynamics of the optimized treatment strategies (1–7) are given in the table.

As previously mentioned, there were eight specific optimized treatment strategies. Of these, seven were found to be effective across all dynamics (figure 10). These were [*t*_dur_, *t*_rec_ (min)] = [10, 20], [10, 30], [20, 50], [20, 60], [20, 70], [20, 80], [20, 90]. On average these strategies reduced the antibiotic dose by 72.2 ± 8.9% across all persistence dynamics. This average is not far from the reduction in antibiotics achieved by specifically optimized treatment dynamics to the given persistence dynamics. However, the spread in effectiveness indicated by the error is larger. This stems from all seven strategies being almost as effective as specific tailored optimums for most persister switching dynamics (*α*_max_ and *β*_max_). But for some persister switching dynamics the difference between specific tailored optimums and these generalized optimums were significant (up to 24.5% less effective). The further a given strategy differed from a given dynamics specific optimum the less effective it was. This meant that the dynamics with shorter optimums (smaller *α*_max_ and *β*_max_) were less well suited to the longer strategies and vice versa.

The most universally successful strategy consisted of 10 min treatments (*t*_dur_) and 30 min recovery (*t*_rec_) which on average across all the persister switching dynamics reduced the required antibiotic dose by 74.9 ± 7.3 (figure 10). As well as performing best on average across all dynamics, it also had the lowest maximum difference of 9.0% from the specific optimum across all the dynamics (electronic supplementary material, figure S3). These results demonstrate how for a given biofilm; universal strategies may exist that are effective regardless of persister dynamics.

### 3.4. Impact of biofilm persistence dynamics on treatment

**Figure 11.**
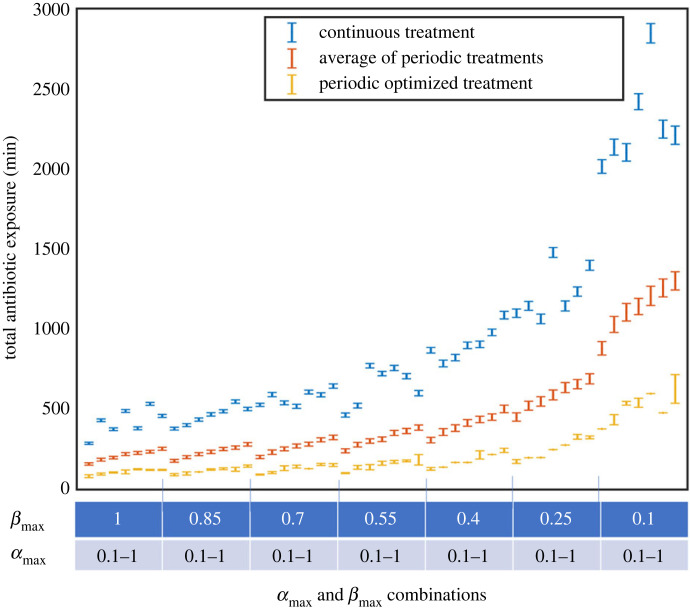
The total antibiotic exposure required to treat biofilms governed by different persistent dynamics. The 49 different persistence dynamics given by combinations of *α*_max_ and *β*_max_ (0.1, 0.25, 0.4, 0.55, 0.7, 0.85, 1) are shown in the table below. Continuous treatments are shown in blue, the average of all tested periodic treatments in red and periodic treatments optimized to the dynamics in yellow.

The biofilm persistence dynamics affect the response to treatments (figure 11). Biofilms with the fastest switching rates from susceptible to persister state and slowest switching rate back required the highest levels of antibiotics. Whereas those with the slowest switching to persister state and fastest switching back to susceptible cells always needed the lowest dose of antibiotics (figure 11). This is true for all treatments, whether continuous or periodic. The difference in the treatment length due to persistence dynamic changes was 2558 ± 398 min equivalent to an 89 ± 14% reduction for continuous treatments and 545 ± 80 min or 86 ± 13% for optimized periodic treatments. The switching rate from the persister to susceptible state had a larger impact on the treatment length than the switching rate from susceptible to persister state (electronic supplementary material, figure S4). On average across all *α*_max_ the change in treatment length due to *β*_max_ was 1860 ± 44 min for continuous treatments and 404 ± 9 min for optimized periodic treatments. Whereas the equivalent changes across all *β*_max_ due to *α*_max_ were 212 ± 24 min and 107 ± 8 min (electronic supplementary material, figure S5).

Biofilms are inherently stochastic and heterogeneous, which can impact their response to treatment. We therefore ran each set of dynamics three times and took averages. We found that across all the treatments and biofilm dynamics tested in this study that there was a standard deviation of 12 ± 8% (electronic supplementary material, figure S6). As seen in electronic supplementary material, figure S7, despite this there were still significant differences between the response of different biofilms and between different treatments.

## 4. Discussion

Persistent subpopulations present a significant challenge in the treatment of bacterial biofilms [4,5]. Periodic pulses of antibiotics could be a viable strategy for sensitizing and treating these subpopulations [27–31]. Our results showed, in line with previous studies [27,28,31], that periodic dosing sensitized persister populations and reduced the total required antibiotic dose. Biofilm experiments can be time consuming and expensive. This in combination with the emergent, heterogeneous nature of biofilms [2,23,33,59] has made determination of effective pulsing strategies challenging [27,29,34]. We developed an agent-based *in silico* model to simulate a wide variety of biofilm, persistence and treatment dynamics rapidly and cheaply.

Our models were used to determine optimized periodic treatment regimens for a range of persistence dynamics. On average implementing treatments with dynamics aligned to the biofilm's dynamics reduced the antibiotic dose by 76.9 ± 0.6%. The optimal duration and recovery times of optimized periodic treatments depended on the biofilm dynamics, but the optimal recovery time (*t*_recop_) was always shorter than the duration (*t*_durop_). This is in line with initial studies that found that *t*_recop_ is shorter than *t*_durop_ with the ratio between the two depending on the biofilm dynamics [31,39].

The effectiveness of optimal treatments was also linked to the persistence dynamics. Optimized periodic treatments were least effective at faster switching rates (higher *α*_max_ and *β*_max_) and most effective at slower switching rates (higher *α*_max_ and *β*_max_). This correlated with optimal periodic treatments dynamics (*t*_recop_ and *t*_durop_). There is a sweet spot for optimal periodic treatment and recovery lengths that minimizes susceptible populations’ regrowth while ensuring adequate ‘reawakening’ of persister populations. In this study at higher *α*_max_ and *β*_max_ the optimal treatment may have been limited by the minimum treatment duration. *In vivo* and *in vitro,* there may be limitations on how quickly antibiotics can be stopped and started, putting restrictions on the achievable length of treatment times. Our results suggest that this would have a larger impact on the treatment of biofilms governed by faster persistence dynamics.

Biofilm and persister dynamics can vary and change. Biofilm pharmacokinetics and dynamics can also be challenging to quantify and monitor, especially in the body. Generalized strategies that are effective against a range of dynamics could provide a powerful solution to this issue. In this work, we found that the specific treatments optimized for one set of persistence dynamics were also effective as generalized treatments. The optimum generalized strategy reduced the required antibiotic load by 74.9 ± 7.3%. Additionally, our observations revealed that across a wide range of persistence dynamics, the periodic dosing regimens we examined consistently lowered the total antibiotic dosage necessary for successful biofilm eradication. This implies that periodic dosing is robust against the diverse dynamics and environments intrinsic to biofilms.

Traditionally persistent subpopulations were overlooked, and antibiotic dosing was based on the dynamics of susceptible cells, leading to ineffective treatments. Persistence may be linked to resistance [16,23,25] it is therefore crucial that treatments fully eliminate all cells. This will prevent the infection recurring as well as the development of antibiotic resistance.

Further determination as to the role of persistence in the emergence of resistance as well as studies into the evolution of resistance under different dosing regimens are required. In the meantime, we must ensure periodic dosing regimens effectively treat the whole biofilms and are robust amongst changing dynamics and environments.

Through the simulation of a wide range of different scenarios and dynamics it is possible to construct a map of the parameter space, as exemplified in this study. This facilitates the assessment of the performance of strategies under varying and unpredictable conditions and dynamics. By using an agent-based model, we were also able to examine the variation in treatment response due to biofilm heterogeneity in biofilms governed by the same parameters. We found a 12 ± 8% standard deviation across the different treatments and dynamics tested. This variation arises due to spatial heterogeneity of cell populations and stochasticity in cell growth, cell death, persister formation and location in the biofilm, which in turn affect persister ‘reawakening’ and the overall response to antibiotics. It has been shown that the spatial arrangement of different cell populations can have a significant impact on how biofilms respond to and recover from antibiotic treatment [4,16,35]. There can be considerable variability in the response of biofilms grown under the same experimental conditions to pulse dosing of antibiotics [27]. It is therefore key that the heterogeneous nature of biofilm is captured by *in silico* models and that strategies are resilient in the face of the diverse dynamics and environments intrinsic to biofilms.

We have focused on looking at whether periodic treatments may be used to target persistent populations in biofilms. This is an initial explorative study. The dynamics and time-scale constraints that may be relevant for a given *in vitro,* or *in vivo* application would need to be included to determine specific strategies for a given scenario. There can be large heterogeneity across different biofilms and scenarios, and so further experimental and clinical parametrization of the pharmacokinetics of a range of biofilm is required to apply our model further. However, the overall results and the models developed in this study are widely applicable. Our model could be used to assess and explore different treatment regimens for a range of *in vivo* and *in vitro* biofilms. The model has been developed to be readily adaptable and widely applicable and is accessible and published in a range of different formats. It can be run with a graphical user interface so that non-technical users can parametrize it for their system, or it can be used to explore the impact of altering various parameters by sliding toggles and visualizing the simulations and outputs. Alternatively, it is available as raw code which can be run more efficiently and is more readily adaptable.

We found that the antibiotic dose required to effectively treat biofilms was linked to the persistence dynamics. Across both continuous and periodic treatment approaches, biofilms with the fastest switching to the persister state and slowest switching back required the highest dose of antibiotics. Conversely, those with the slowest switching to persister cells and fastest switching back to susceptible cells always required the lowest dose. Several strategies may be used in combination with periodic dosing to change the switching dynamics of the biofilm and reduce the antibiotic dose further. These involve inhibiting persister formation or waking them up [34,52,60]. These changes would correspond to a decrease in the rate of persistence formation (lower *α*_max_) or increase in switching rate back to susceptible cells (higher *β*_max_). The results from our model suggest that decreases in persistence formation and/or an increase in persistence awakening could increase the effectiveness of implementing just periodic treatments by an extra 20% (figure 11).

In the future, *in vitro* and *in vivo* work is required to test this hypothesis and the effectiveness of combining strategies. Further work is also required to understand the mechanisms underpinning persistence and to quantify persistence dynamics in more diverse biofilms. As new data related to experimental parametrization and biological understanding emerges, our models can be used for a range of biofilms and applications.

Recent advances in how we deliver antibiotics could also allow more efficient dosing strategies, such as those explored in this study, to become a reality. For example, microarray patches have been developed that allow an easily controllable and accurate way of delivering antibiotics directly into the blood stream or a biofilm [61,62]. Ultimately, there is potential to combine this approach with real-time infection monitoring [63] and extract biofilm parameters for treatment optimization. In the future, it may be possible to adopt a closed-loop methodology for infection management or to tailor treatments to individual patients. *In silico* models offer a powerful tool for developing and implementing these strategies.

## 5. Conclusion

Consistent with prior research, our findings highlight the potential of periodic dosing to ‘reawaken’ persister cells and diminish the overall antibiotic dosage needed to treat a biofilm.

We developed an agent-based *in silico* model to rapidly explore a wide parameter space and range of treatments. Our model can be used to obtain tailored optimal dosing regimens for a range of persistence dynamics. Furthermore, it can be used to derive generalized optimal dosing regimens that effectively reduce the total antibiotic burden across different dynamics.

Our study also revealed that the optimized dosing regimens dynamics were contingent upon persister formation dynamics. However, we did manage to identify a single dosing regimen that remained effective across a broad array of persister switching rates. Looking forward, further parametrization of the pharmacokinetics and pharmacodynamics parameters for distinct biofilms, antibiotics and persister formations will empower us to tailor dosing strategies to different systems and offer routes to tackling the pressing issue of AMR.

## Data Availability

The Netlogo model and supporting code is available from the Zenodo repository: https://zenodo.org/doi/10.5281/zenodo.10731490 [64]. Supplementary material is available online [65].
