# Correction: ‘Optimization of periodic treatment strategies for bacterial biofilms using an agent-based *in silico* approach’ (2024) by Blee *et al.*

**DOI:** 10.1098/rsif.2025.0509

**Published:** 2025-07-16

**Authors:** Johanna Blee, Thomas Gorochowski, Sabine Hauert

**Affiliations:** ^1^ School of Engineering Mathematics and Technology, University of Bristol, Bristol BS8 1TW, UK; ^2^ School of Biological Sciences, University of Bristol, Bristol, UK

*J. R. Soc. Interface* **21**: 2120240078 (published online 10 April 2024). (http://doi.org/10.1098/rsif.2024.0078)

In the Funding statement, the EPSRC grant code EP/T517872 is incorrect. The correct grant code is EP/T517872/1.

